# Diagnosis and treatment of retroperitoneal bronchogenic cysts: A case report

**DOI:** 10.3892/ol.2014.1974

**Published:** 2014-03-14

**Authors:** BIAO DONG, HONGLAN ZHOU, JIANJIAN ZHANG, YUANTAO WANG, YAOWEN FU

**Affiliations:** 1 Department of Urology, First Hospital of Jilin University, Changchun, Jilin 130021, P.R. China; 2 Department of Hepatic Surgery, Renji Hospital, Shanghai Jiaotong University School of Medicine, Shanghai 200127, P.R. China

**Keywords:** bronchogenic cysts, retroperitoneal

## Abstract

Bronchogenic cysts are uncommon, predominantly benign, congenital malformations arising from the primitive foregut. The occurrence of such cysts in the retroperitoneum is extremely rare. The present study presents the case of a 30-year-old female who presented with a left adrenal mass. Imaging investigations revealed a cystic mass located medially to the left adrenal gland. Retroperitoneal laparoscopic excision and complete resection were performed, and the subsequent pathological examination confirmed the diagnosis of a bronchogenic cyst in the retroperitoneum. The patient was discharged on the fourth post-operative day and received no further treatment, however, regular follow-up was performed due to the lesion being benign. A rare case of bronchogenic cyst and literature review is presented, which may aid in improving the understanding of the etiology and pathogenesis of retroperitoneum bronchogenic cysts.

## Introduction

Bronchogenic cysts are foregut-derived congenital abnormalities that occur following the third week of embryonic life (1). When attached to the primitive foregut, the cyst is usually associated with the tracheobronchial tree. However, in rare instances, the cyst may separate from the normal airways, presumably by migration, and thus, may be found in atypical locations, including the neck, intraspinal locations and below the diaphragm (2). Bronchogenic cysts presenting in the abdomen or retroperitoneum have rarely been reported in the medical literature. Furthermore, as bronchogenic cysts are usually asymptomatic, unless they become secondarily infected or enlarge enough to compress adjacent organs, the majority of reported cases are diagnosed incidentally (3,4). The current study presents the case of a bronchogenic cyst in the retroperitoneum of a 30-year-old female, which was successfully managed by retroperitoneal laparoscopic surgery. In addition, the clinical, radiographic, surgical and pathological observations are summarized. Patient provided written informed consent.

## Case report

**Figure 1:**
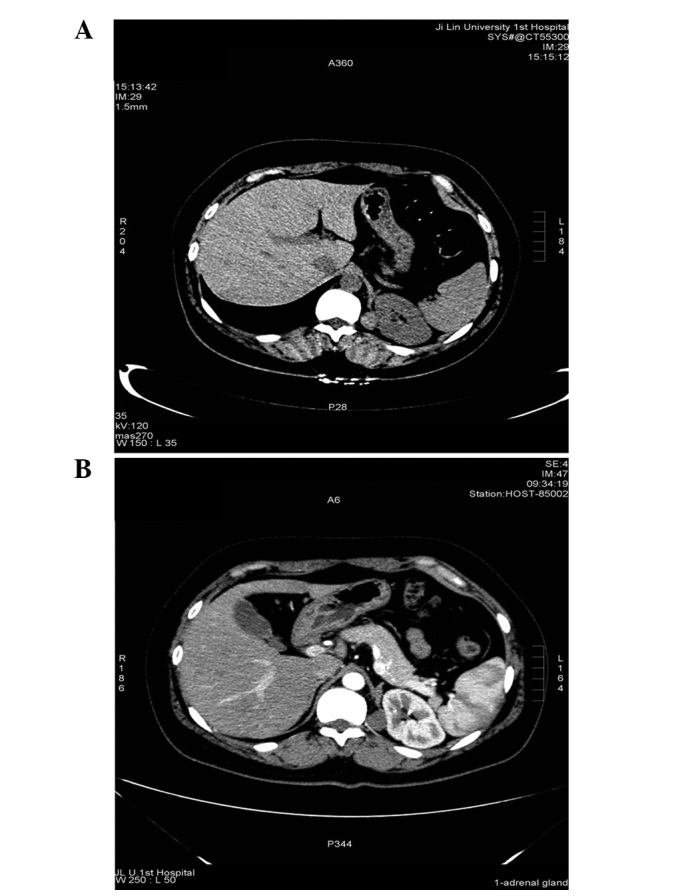
(A) Computed tomography (CT) scan revealing a cystic mass in the retroperitoneal area located medially to the left adrenal gland. (B) Contrast enhanced CT scan revealing a homogeneously dense cystic mass in the left suprarenal region.

A 30-year-old female was referred to the First Hospital of Jilin University (Changchun, China) for the evaluation of a left adrenal mass, which was identified incidentally during an examination for a persistent fever, associated with coughing and expectoration lasting for three days. A computed tomography (CT) scan of the abdomen revealed a cystic mass measuring 32 Hounsfield units, with a diameter of ~1.9 cm, located medially to the left adrenal gland. However, the cystic mass was not clearly demarcated from the upper pole of the left kidney (Fig. 1A). An enhanced CT scan revealed a homogeneous, round, low-density mass with smooth outlines situated in the retroperitoneum and slight enhancement (Fig. 1B).

**Figure 2:**
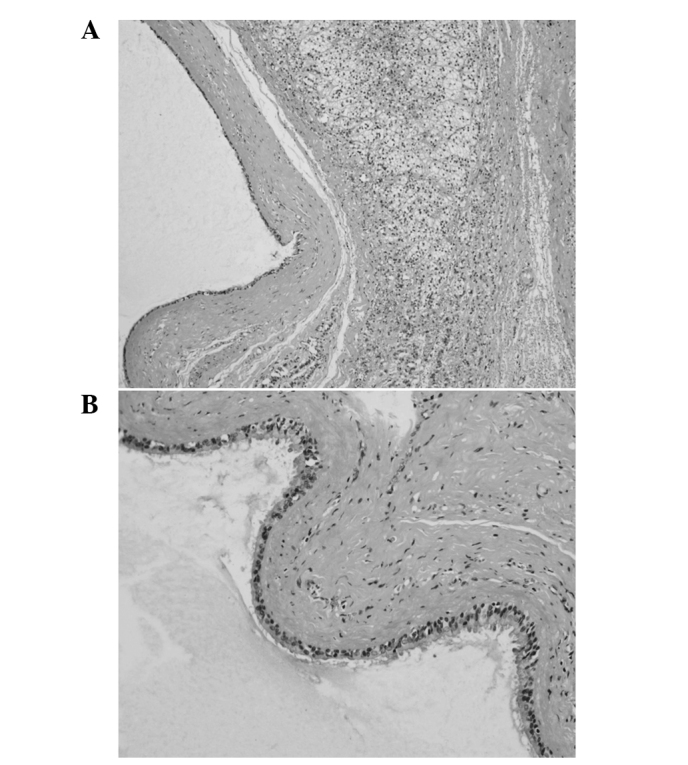
(A) Histological appearance of the specimen revealing a ciliated pseudostratified columnar epithelium and diagnosed as a bronchogenic cyst (hematoxylin and eosin staining; magnification, ×100). (B) High power magnification of the cyst revealing a lining of a pseudostratified or tall columnar ciliated epithelium (hematoxylin and eosin staining; magnification, ×200).

The patient had no palpitations, diaphoresis, weakness or hypertension. In addition, no other significant medical history was noted. The patient’s endocrine evaluation results for adrenal hypersecretion were negative, and the results of testing for the renin ratio, aldosterone, plasma and urinary catecholamines and metanephrines were all within the normal ranges. The patient was admitted for surgical removal of the mass, and the laparoscopic surgery revealed a 1.5×2.0×2.0-cm cystic structure with a complete capsule, which was adherent to the upper pole of the left kidney. The cyst was located deep within the retroperitoneum in the immediate vicinity of the medial margin of the adrenal gland and adjacent tissue. The pathological evaluation revealed a cystic mass filled with a white seromucinous fluid. In addition, the histopathological examination revealed that the cyst was partially lined with ciliated pseudostratified epithelium, with the cyst wall containing a small number of seromucous glands (Fig. 2).

The patient had an uneventful post-operative recovery and was discharged on the fourth post-operative day. The patient received no further treatment, however, regular follow-up was performed due to the lesion being benign.

## Discussion

When the tracheobronchial tree undergoes abnormal budding and pinching off at approximately week five of gestation, bronchogenic cysts develop (1). If the connection with the tracheobronchial tree is lost, the foregut and its derivatives that are usually in close proximity to the trachea and bronchus may migrate to an atypical location (5). Retroperitoneally-located bronchogenic cysts may occur as the pericardioperitoneal canal links the thoracic and abdominal cavities (6).

Although bronchogenic cysts are rare, such cysts must be considered in the diagnosis of a retroperitoneal mass. However, the pre-operative diagnosis remains a clinical issue. Retroperitoneal bronchogenic cysts may easily be misdiagnosed as adrenal cortical or medullary tumors, or enteric, urothelial or pancreatic cysts by their clinical and radiological presentation. However, a histological diagnosis may differentially determine a bronchogenic cyst from such lesions. Microscopically, bronchogenic cysts are predominantly unilocular or oligolocular, lined by pseudostratified ciliated columnar epithelium with bronchial glands, mucoid material, cartilage and smooth muscle (7,8). In the present study, the patient was diagnosed with a bronchogenic cyst based on the presentation of the following histological features: A ciliated pseudostratified epithelium and a small number of seromucous glands.

The mainstay of treatment for retroperitoneal bronchogenic cysts is surgical removal. Although the majority of cysts are asymptomatic and exhibit benign behavior, excision is recommended to establish a diagnosis, to alleviate any symptoms and to prevent infection and the documented risk of malignant transformation (9). According to the literature, laparoscopic excision has been widely used to treat retroperitoneal bronchogenic cysts (10,11). Furthermore, as this approach uses small incisions, it has the potential to decrease the duration of hospitalization and therefore, reduce costs to the hospital and patient.

In conclusion, the current study presents a rare case of a retroperitoneally localized bronchogenic cyst as an unusual differential diagnosis of a retroperitoneal tumor. The combination of clinical, biochemical and radiological features may aid in the characterization of lesions, however, only a histological analysis can currently provide a definite diagnosis.
